# The Queen's Hall Meeting: The Real Significance of the Demonstration

**Published:** 1911-12-23

**Authors:** 


### THE QUEEN'S HALL MEETING.

### The Real Significance of the Demonstration.

BY AN M.D. WHO WAS PRESENT.

For pure intellect Sir Victor Horsley has admittedly not many equals, and perhaps no superiors, in the medical profession. It would not be very surprising to find that Sir Victor in his own mind regards ninety-nine hundredths of his fellow practitioners as fools; and is as little moved by their opinions about himself or about medical politics as he is by the opinions of Fiji Islanders about cranial surgery. Yet for all that Sir Victor Horsley would do well to bear in mind a famous saying of a more able man of affairs, if a much less distinguished intellectualist, than himself—namely, Abraham Lincoln. That statesman was wont to say, "You can fool some of the people all the time, and you can fool all the people some of the time; but you can't fool all the people all the time." For the consensus of opinion among the profession is that the Council of the British Medical Association, on which body Sir Victor Horsley is the most outstanding personality, has tried to run with the hare and hunt with the hounds; and has, in fact, betrayed the trust reposed in it by the profession at large and the Association's members in particular.

It is all very well for Sir Victor and Drs. Keay and Rice Oxley to justify their actions by quotations from minute-books and similar documents, as they did in the communication issued to the Press after the Queen's Hall meeting. Whether or not they have been technically correct, the general feeling of the profession is that they have not secured the six-point programme, and that they have sacrificed the profession on the altar of party politics and party loyalty. That is the real meaning of the refusal of the meeting to hear Sir Victor's explanations, of the shouts of "Traitor" and "Judas" hurled at him by staid and middle-aged practitioners from every quarter of the Queen's Hall. He has overreached himself through forgetting that "You cannot fool all the people all the time."

### Impressions of the Meeting Itself.

About the success of the meeting there could be no two opinions. The large hall at Langham Place was packed on the ground floor, crowded on the first gallery, and a few even ascended to the second gallery to get a good view. The audience consisted almost entirely of medical men, mostly of middle age at least. The number of medical students present was infinitesimal, and the endeavours of the "Daily Chronicle" to represent the contrary is a falsification of the facts. Nor was there much doubt about the unanimity of those present. A few sympathisers the Council of the British Medical Association had, but not more than seven or eight hands were held up against the first resolution, and even fewer against the second one.

In Sir Watson Cheyne an admirable chairman had been secured. It was through no fault of his that the meeting got out of hand at times. His introductory speech was excellent alike in tone, in substance, and in delivery. Especially noteworthy was his exhortation to those present not to follow his example (set a few years ago) of leaving the Association. He emphasised the facts that organisation is essential, and that the B.M.A. already has both that and the necessary funds without which organisation cannot be translated into action.

The first resolution was proposed by Dr. F. J. Smith, whose quiet but brief and telling sentences put the whole case against the Council of the B.M.A. in a nutshell. Needless to say, as the convener of the meeting Dr. Smith had a tremendous reception; and he rose to the occasion as everyone who knows him was sure that he would. Dr. Smith is not the sort of orator who can fire a crowd by mere oratory; but the studied precision of his indictment and the calm, forceful way in which it was presented struck exactly the right note. Unfortunately, subsequent orators did not always follow Dr. Smith's example, though we are not suggesting that any lack of restraint on their part had any great effect in causing the disorderly scenes which ensued later. The seconder of the first resolution was Mr. M. M. Townsend. Evidently feeling deeply on the subject under discussion, he might have imitated the quieter oratory of Dr. Smith, though it was evident that his speech was highly to the taste of many of his audience.

### The Scene.

Then Sir Victor Horsley rose in the body of the hall, and the scene began. Invited by Sir W. Cheyne to ascend the platform, Sir Victor made his way there amidst a perfect storm of hisses, booing, and opprobrious epithets; and when he attempted to address the meeting the tumult became worse than ever. Whatever else Sir Victor may be, he is a man not easily daunted; and he faced the hostile meeting with the utmost *sangfroid*. The Chairman did his utmost to secure a hearing for Sir Victor, and the stewards also did everything possible to soothe the handful of irreconcilables who continued the uproar. But it was no good, and at last the speaker had to accept the inevitable and sit down, a proceeding followed by a thunderous outburst of cheers.

The second resolution was moved in a speech which excited a good deal of merriment among the more thoughtful of those present. Before this gentleman addresses a big meeting again he would do well to avoid composing a harangue in the manner of a stump orator, and delivering it in the manner of a provincial actor. But some of the subsequent speeches were first-class, notably those of the seconder, Dr. Helme, and of Dr. Pinder. Disorder broke out again when Dr. Keay began to speak; and before he had been very long over his defence of the Council of the

British Medical Association the clamour was such that he also had to retire with his speech unfinished.

### The Upshot.

Of course, matters will not and cannot rest at this point. As Dr. Smith announced, the meeting is only the preliminary to further concerted action; and clearly the first step will be the reconstitution of the Council of the British Medical Association. It cannot be imagined that that body will continue in office in defiance of the practical unanimity of the members that it must resign. Here, then, the Association will be faced with an internal crisis, though it is evident that with a reconstituted Council it must emerge far more potent in defence of the threatened interests of its members than it has been during the passage of the Insurance Act.

It is clear that the members of the Association must bear part of the blame for electing a Council which could not be relied on to carry out its wishes. It is all very well to turn and rend the Council now, but the truth is that several members of the Council should never have been elected to it. Sir Victor Horsley is, as everyone who takes any interest in public affairs knows, a very advanced Radical in politics and one who has himself ambitions of a parliamentary career. Was it likely that such a representative would deal with Mr. Lloyd George except with a strong bias towards that gentleman's schemes? Dr. F. J. Smith hinted pretty broadly that the two pounds a week limit could have been secured had not the negotiators on behalf of the medical profession been afraid of upsetting the Government. We presume that Dr. Smith had solid grounds for this statement, and we are not very much astonished at it. The fact of the matter is, the members of the Association have allowed their affairs to get into the hands of a set the most energetic and pushful of whom are extreme Radicals; and the natural result is that in dealing with a Radical Party in power the Association starts under a heavy handicap.

### The Insurance Commissionership.

Then, again, the appointment of Mr. Whitaker could hardly fail to come in for animadversion at the Queen's Hall. The real meaning of this appointment is, of course, that it is simply a move in the game of one of the most astute politicians who have held power in this country. Yet here, again, it is possible to be too astute; and the plain fact is that, after successfully bamboozling the profession through the medium of the Council of the British Medical Association, the Chancellor has by this appointment made a mistake which is likely to cost him dearly. For he has aroused the profession from the slumber so skilfully induced by his dealings with the Council, and now the fat is in the fire. Furthermore, the appointment is unsound in other ways. To safeguard the interests of the profession on the Insurance Commission will be no easy task, and the profession at large has no confidence in the ability of the late medical secretary of the British Medical Association to grapple with this task.

### A Further Proof.

If any further proof were required of the feelings of the profession beyond the extraordinary success of the Queen's Hall meeting and of the great meeting in Manchester a few days previously, it is amply furnished by the results of the referendum promoted by *The Practitioner*. Our contemporary sent out voting-papers to the twenty-nine thousand odd members of the profession in Great Britain. The object was to discover whether or no the Act as it now stands is satisfactory to the profession. It will be seen that this is tantamount to asking whether the "six-point policy" of the British Medical Association has been effectively secured or guaranteed. For it was decided long ago that the Act (or Bill, as it then was) would be satisfactory if those points were conceded. The result of the census is very striking. Over 20,000 answers were received, and for 352 to whom the Act is satisfactory there are 20,149 to whom it is not satisfactory and 211 who have not decided. Such unanimity of opinion is extraordinary, considering the very varied interests of the numerous sections into which the medical profession is to-day sub-divided. In itself this constitutes a vote of censure such as the Council of the Association dare not disregard.

### The Act Itself.

What bearing have all these indications of the sentiments of the profession upon the Insurance Act itself? To begin with, there are indications that the Act is so unpopular that it would not cause any very great surprise if an amending Act were found necessary before it ever comes into operation at all. The commercial and working classes dislike it, and have been saying so in no measured terms. The hospitals are organising to get its provisions altered, for their very existence is threatened by its provisions as they at present stand. The servants tax is so highly unpopular with both mistresses and maids that a league for resisting it has already obtained a large membership.

On top of all this comes the news that the doctors are dissatisfied, and that their opposition, believed to be lulled, is more determined, because more apprehensive, than it was before. The conclusion cannot be resisted that this ill-digested measure, many parts of which have never been debated at all, must be either abandoned altogether or else re-discussed and reformed from one end to the other. If it has done nothing else, it has united the medical profession, and the great Queen's Hall meeting is only the outward sign of that unity. To its promoter, Dr. F. J. Smith, the thanks of the profession are due, and we do not fancy that he will ever have cause to regret his action in convening it.

